# The 11-Year Prognostic Impact of Chronic Total Occlusion in the Noninfarct-Related Coronary Artery on Patients with Acute Myocardial Infarction

**DOI:** 10.1155/2021/6646804

**Published:** 2021-03-17

**Authors:** Xuanqi An, Jingang Yang, Kefei Dou, Yuejin Yang

**Affiliations:** Department of Cardiology, National Center for Cardiovascular Diseases, Fuwai Hospital, Chinese Academy of Medical Sciences and Peking Union Medical College, No. 167, North Lishi Road, Xicheng District, Beijing 100037, China

## Abstract

**Background:**

The prognostic significance of CTO in the non-IRA in patients with AMI has been under dispute. Relevant long-term follow-up studies are lacking. *Hypothesis*. CTO in the non-IRA is an independent predictor of poor long-term prognosis in patients with AMI.

**Methods:**

We prospectively enrolled 2336 patients with AMI who received emergent percutaneous coronary intervention successfully from January 2006 to May 2011. Our primary endpoints included death from cardiovascular causes, recurrent myocardial infarction, stroke, and target-vessel revascularization. We adopted Cox regression analysis adjusted for confounders to analyze the impact of CTO in the non-IRA on long-term mortalities.

**Results:**

We identified 628 (27.6%) subjects with CTO in the non-IRA among 2282 AMI patients. After a mean follow-up duration of 134.3 months, we found the CTO group had significantly higher MACCE rate than the group without CTO (30.4% versus 24.3%, *P*=0.004). CTO in the non-IRA independently predicted 11-year MACCE in the male AMI subgroup (hazard ratio 1.28, 95% confidence interval 1.06 to 1.54, *P*=0.01) and in the male NSTEMI subgroup (hazard ratio 1.53, 95% confidence interval 1.09 to 2.15, *P*=0.02). In the CTO group, three-vessel disease independently predicted 11 year MACCE (hazard ratio 2.05, 95% confidence interval 1.29 to 3.28, *P*=0.002).

**Conclusions:**

Our long-term observational study supported the association between CTO in the non-IRA and poorer prognosis in AMI patients undergoing primary PCI. We identified the group with the three-vessel disease as a high-risk subgroup in patients with CTO in the non-IRA.

## 1. Introduction

Acute myocardial infarction (AMI) is the primary cause of death in patients with coronary heart disease (CHD). The introduction of percutaneous coronary intervention (PCI) has identified chronic total occlusion (CTO) of the noninfarct related artery in about 10–30% patients with AMI [1, 2]. Opinions divide on the prognostic impact of CTO in the non-IRA on the patients with AMI due to conflicting results produced by different follow-up studies [2–5]. Also, the longest follow-up duration is 6 years, and the majority of the study population confines to patients with ST-segment elevation myocardial infarction (STEMI) [6, 7]. Our study aims to evaluate the effects of CTO in the non-IRA on the patients' total mortality with STEMI or non-ST-segment elevation myocardial infarction (NSTEMI) 11 years after PCI through a long-term follow-up.

## 2. Materials and Methods

### 2.1. Study Population

The Ethics Committee in our institution has approved our study. From January 2006 till May 2011, we prospectively enrolled patients diagnosed with AMI who received emergent PCI within 24 hours after infarction successfully at our emergency department. We diagnosed STEMI if patients exhibited symptoms of myocardial ischemia with elevated serum troponin value ≥ two times the upper limit of normal and evolving electrocardiographic (ECG) ST-segment elevation more than 1 mm (0.1 mv) or new left bundle branch block in more than two contiguous leads. We considered NSTEMI when the patient manifested symptoms suggestive of myocardial ischemia with elevated serum troponin value ≥ two times the upper limit of the normal range. Furthermore, we confirmed the diagnosis of AMI through the emergent coronary angiography for all patients. We excluded patients with AMI with previous coronary bypass surgery to avoid the potentially confounding impact of different vessel anatomies. We also rejected patients with unsuccessful primary PCI or cardiogenic shock on admission because these factors could contribute to the mortality tremendously and might influence our analysis. Apart from signing standard informed consent regarding both the disease and the interventional procedure, all patients have agreed to participate in the study after they were fully informed about the follow-up plan. We recorded their baseline characteristics, including age, sex, ID number, contacts information, relevant risk factors, smoking habits, left ventricular ejection fraction measured by echocardiography, medications, and lab test results such as hemoglobin levels, cardiac enzymes, and liver and kidney functions both before and after the procedures. Before the intervention, we prescribed 300 mg aspirin and 300 mg clopidogrel or 180 mg ticagrelor to all patients as the loading doses.

### 2.2. Percutaneous Coronary Intervention

Experienced interventional cardiologists on duties performed the emergent coronary angiographies. They chose different arterial puncture sites, guidewires, catheters, inflation balloons, coronary stent types, and intravascular ultrasound or glycoprotein IIb/IIIa inhibitors based on the technical standards of the time and ongoing clinical conditions. We adopted the Thrombolysis in Myocardial Infarction (TIMI) scale for assessing coronary blood flow. IRA was determined according to the ECG manifestations and the typical angiographic images. We defined multiple vessel disease (MVD) as ≥70% diameter stenosis in one or more major epicardial arteries or major branches. We considered CTO in the non-IRA if the non-IRA was completely occluded (TIMI 0 blood flow) with angiographical features of chronically occluded arteries such as filling through collateral circulations. In all patients, only IRA was revascularized, and drug-eluted stents were implemented instead of bare-metal stents. We regarded the procedure's success if TIMI ≥ 2 was restored on the IRA and resolution of the patient's symptoms. The operators removed the vascular sheath after coagulation parameters normalized. Moreover, we collected their angiography results and procedure data while we further excluded the patients with unsuccessful intervention.

### 2.3. Medical Treatments

After interventions, all individuals received guideline-based medical therapies during hospitalization and follow-up periods. We implemented one year of dual antiplatelet therapy, including aspirin and clopidogrel or ticagrelor. Beyond one year, we recommended daily aspirin for all patients. We also initiated high-dose statin, beta-blockers, angiotensin-converting-enzyme inhibitors, or angiotensin-receptor blockers for all patients unless contraindicated.

### 2.4. Follow-Up Plan and Study Endpoints

Our study's primary endpoint was Major Adverse Cardiovascular and Cerebrovascular Events (MACCE) at 11-year follow-up, including death from cardiovascular causes, recurrent myocardial infarction, stroke, and target vessel revascularization. Cardiac death was defined as death due to an AMI, fatal arrhythmia, or exacerbated heart failure (HF). Having measured the in-hospital MACCE among these patients, we contacted the patients by telephone and home visits during the follow-up period.

### 2.5. Statistical Analysis

We determined our sample size by implementing the rule of thumb: matching observed variables with the observed study population. It mandated our enrollment number was 10 to 20 times larger than the included variables. Since we decided to include 15 variables in the regression analysis, and the 11-year mortality for AMI patients is around 15%, we estimated the included individuals of 2000 (15*∗*20/15%). Moreover, considering a 10% loss of follow-up, we determined our sample size of 2223 individuals. We consecutively recruited 2336 AMI patients.

We presented continuous variables as mean ± standard deviation if they were normally distributed or as median (interquartile range) if they were not. The Shapiro–Wilk test was used to assess the normality of distribution. A *P* value <0.05 was considered to indicate statistical significance. We compared continuous variables with Student's *t*-test or the Wilcoxon rank-sum test based on their distributions. For categorical variables, we adopted numbers and percentages to present them, while we utilized *v*2 test or Fisher's exact test to compare them when appropriate. We employed the Kaplan–Meier method to estimate the cumulative incidences of clinical events and evaluated the differences with the log-rank test. We utilized a multivariable Cox proportional hazards model to explore the effect of CTO in non-IRA on MACCE during the entire follow-up period. We entered multiple potential confounders in the regression model, including age, sex, cardiovascular risk factors (previous history of hypertension, diabetes, hyperlipidemia, and stroke), smoking history, the severity of CAD (types of lesions, numbers of the vessel affected), and major adverse cardiovascular events after initial coronary revascularization at the first hospitalization.

## 3. Results

### 3.1. Patients, Treatments, and Follow-Up

From January 2006 till May 2011, we consecutively enrolled 2336 patients diagnosed with AMI (STEMI or NSTEMI) in our medical center's emergency department. They all underwent emergent revascularization of IRA within 24 hours after infarction. We excluded ten patients for unsuccessful interventions and further rejected seven patients who presented with cardiogenic shock on admission. We subsequently assessed the outcome events by outpatient visits or telephone calls if they declined to visit our outpatient center, and our final follow-up visit dates ranged from November 2019 to February 2020. Our mean follow-up interval was 134.3 months, during which we lost contact with 37 patients. We included 2282 AMI patients with their baseline characteristics and angiographic data in our final analysis and divided them into two groups: the group of STEMI patients and the group of NSTEMI patients. As noted in Table 1, we identified 682 (27.6%) patients with concurrent CTO in the non-IRA out of 2282 patients, with 463 (28.0%) in the STEMI group and 165 patients (26.2%) in the NSTEMI group. Compared with the patients in the NSTEMI group, patients in the STEMI group were younger, were predominantly male, were more active smokers, and had relatively lower left ventricular ejection fraction (LVEF) measured by echocardiography. We also grouped them by concomitant CTO in the non-IRA and compared their baseline parameters in Table 2. We discovered patients burdened with CTO were inclined to have significantly lower LVEF (56.4% versus 58.5%, *P* < 0.001), more previous MI episodes (41.9% versus 36.7%, *P*=0.021), increased prevalence of three-vessel disease (50.9% versus 39.7%, *P* < 0.001), and predominantly type C lesions (81.3% versus 50.3%, *P* < 0.001) than patients without, while both groups shared similar profiles including age, sex, and risk factors for cardiovascular diseases. Moreover, the incidence of in-hospital MACCE was significantly higher in the CTO group (4% versus 1.8%, *P*=0.003), owing to the increased target-vessel revascularizations of IRA (21 cases, 3.3% versus 16 cases, 1.0%, *P* < 0.001).

### 3.2. Primary Outcomes

During a mean follow-up interval of 134.3 months, we recorded a total of 125 deaths (5.4%) from cardiovascular causes in our 2282 AMI population. We also observed 157 recurrent AMI episodes, 48 strokes, and 397 target vessel revascularizations (including 60 non-IRA CTO revascularizations). Both the STEMI and NSTEMI groups shared similar characteristics on the aspect of primary outcomes. However, as detailed in Table 3, the group with CTO in the non-IRA had significantly increased primary endpoint rates than the group without CTO (30.4% versus 24.3%, *P*=0.004). The incidence of deaths from cardiovascular causes was higher in the CTO group (43 deaths, 6.8%) than the group without CTO (82 deaths, 5.0%, hazard ratio = 1.36; *P*=0.0778). Notably, the percentage of target vessel revascularizations was significantly higher in the group with CTO (127 revascularizations, 20.2%) than in the group without CTO (270 revascularizations, 16.3%, hazard ratio = 1.24; *P*=0.0292). The incidence of recurrent AMI and strokes were also higher in the group with CTO without statistical significance. Within the group with CTO, we identified patients with the three-vessel disease or left main plus three-vessel disease had significantly higher rates of myocardial infarction, strokes, and target vascular revascularizations than patients with the two-vessel disease or single-vessel disease.

### 3.3. Survival Analysis

**Figure 1:**
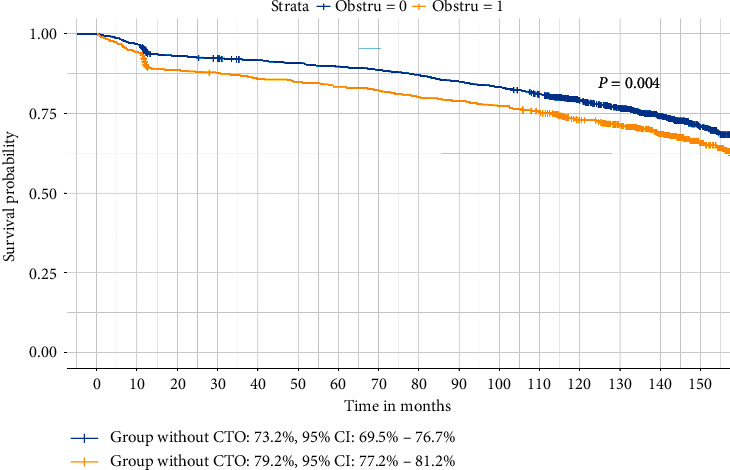
Kaplan–Meier survival curve of all patients with acute myocardial infarction divided by concurrent chronic total occlusion in the non-infarct-related artery. Plot of survival functions in patients with chronic total occlusion versus patients without chronic total occlusion.

The MACCE-free rate at 11 years' follow-up was 77.5% (95% confidence interval 75.8% to 79.3%) in the entire study population, 78.4% (95% confidence interval 76.3% to 80.5%) in the STEMI group, and 75.3% (95% confidence interval 71.9% to 78.9%) in the NSTEMI group. As illustrated in Figure 1, We noticed the MACCE-free rate was significantly lower in the group with CTO in the non-IRA (73.2%, 95% confidence interval, 69.5% to 76.7%) than the group without CTO (79.2%, 95% confidence interval, 77.2%–81.2%, *P*=0.004). We further discovered the MACCE-free rate difference between CTO group and group without CTO was more pronounced in NSTEMI subgroup (69.7% in the CTO group versus 77.4% in the non-CTO group, *P*=0.012) than in the STEMI subgroup (74.9% in the CTO group versus 79.2% in the non-CTO group, *P*=0.061).

### 3.4. Regression Analysis

Monofactor regression analysis for the entire study population discovered CTO in the non-IRA as a strong factor affecting MACCE (hazard ratio 1.29, 95% confidence interval 1.09 to 1.53, *P*=0.004). Prior MI (hazard ratio 1.17, 95% confidence interval 1.00 to 1.38, *P*=0.049), in-hospital MACCE (hazard ratio 1.87, 95% confidence interval 1.25 to 2.76, *P*=0.005), two-vessel disease (hazard ratio 1.36, 95% confidence interval 1.06 to 1.75, *P*=0.01) posed threats while LVEF served as a protective role (hazard ratio 0.98, 95% confidence interval 0.97 to 0.99, *P*=0.005). However, multivariable analysis in Table 4 only showed in-hospital MACCE (hazard ratio 1.85, 95% confidence interval 1.18 to 2.91, *P*=0.008), two-vessel disease (hazard ratio 1.35, 95% confidence interval 1.04 to 1.75, *P*=0.02), and three-vessel disease (hazard ratio 1.59, 95% confidence interval 1.25 to 2.03, *P*=0.0001) were an independent predictor of 11-year MACCE in the entire study population. LVEF remained a protective factor for the 11-year primary endpoints (hazard ratio 0.98, 95% CI 0.97 to 0.99, *P*=0.003).

Regarding the possible role of CTO in the non-IRA in predicting the mortality in the subgroup population, we further performed multivariable regression analysis towards MACCE stratified by sex, STEMI, or NSTEMI. We discovered that CTO in the non-IRA was an independent predictor of 11-year MACCE in the male AMI subgroup (hazard ratio 1.28, 95% confidence interval 1.06 to 1.54, *P*=0.01) and in the male NSTEMI subgroup (hazard ratio 1.53, 95% confidence interval 1.09 to 2.15, *P*=0.02).

Finally, to identify risk factors in the AMI patients with CTO in the non-IRA, we conducted multivariable regression analysis within the CTO group. We concluded that three-vessel disease could independently predict 11-year MACCE for AMI patients with CTO in the non-IRA (hazard ratio 2.05, 95% confidence interval 1.29 to 3.28, *P*=0.002).

## 4. Discussion

The present study aimed to explore the prognostic effects of CTO in the non-IRA on the long-term outcomes of AMI patients who successfully received emergent revascularizations of IRA. And our 11-year follow-up study on 2282 AMI population found patients with CTO had significantly higher rates of target vessel revascularizations. These patients also had increased incidences of deaths from cardiovascular causes, recurrent AMI, and strokes. In the CTO group, we also concluded that the subgroups with the three-vessel disease or left main plus three-vessel disease had significantly increased rates of AMI, strokes, and target vessels revascularizations. We then discovered that the MACCE-free rates in the CTO group were significantly lower than those without CTO in the non-IRA. Last but not least, we supported CTO in the non-IRA acted as an independent predictor of 11-year MACCE in male AMI population. Furthermore, in the CTO group, we identified that MVD could independently predict 11-year MACCE.

CTO remains as the most challenging coronary lesion for PCI and requires optimal operating skills [8]. Data showed the prevalence of concurrent CTO in the non-IRA in STEMI patients varies from 4.7% to 31.5%, while its prevalence in NSTEMI patients ranged from 7.1% to 47% [7, 9–11]. Recanalizing CTO in the non-IRA for AMI patients remains under debated due to limited and conflicted data on the effects of CTO in the non-IRA on the long-term prognosis of AMI patients. Tajstra et al. first conducted a 5-year follow-up study on 1658 STEMI patients and concluded that CTO in the non-IRA was an independent predictor of mortality [7]. Gierlotka et al. analyzed outcomes of 925 NSTEMI patients and found CTO in the non-IRA significantly correlated with 1-year mortality [11]. However, Lee et al. reported that CTO in the non-IRA was not a significant predictor of 1-year mortality on 1008 AMI patients [12]. Similarly, Ariza-Sole et al. contended CTO in the non-IRA did not act as an independent predictor of 1-year mortality on 1176 STEMI patients [2]. Lesiak et al. performed the longest 6-year follow-up study on 836 STEMI patients and supported CTO in the non-IRA's predictive role [6]. Last but not least, O'Connor et al. carried out a meta-analysis encompassing 11451 STEMI patients from 7 observational studies and showed CTO in the non-IRA was independently associated with increased mortality at a mean follow-up of 25.2 months [13]. We believe the possible etiologies for such conflicting results may lie in the following areas. (1) Prevalence of CTO in the non-IRA varied greatly across study populations. These differences might reflect diversified baseline microcirculation status, which could contribute to a distinct long-term prognosis. (2) There were different sample sizes and follow-up intervals. (3) Diverse mortality rates ranged from 11.2% to 38.6% across studies, which might indicate underlying differences in the study populations and treatment regimens. (4) Different studies adopted different variables for regression analysis.

Other than observational studies mentioned above, some studies, including randomized controlled trials, aim to explore the prognostic effect of recanalizing CTO in the non-IRA for AMI patients. Yoshida et al. performed a comprehensive 10-year follow-up study on 1184 AMI patients and testified recanalizing CTO in the non-IRA had significant benefits [14]. Choi et al. conducted a 5-year follow-up study on 4748 AMI patients and supported the protective effects of recanalizing CTO in the non-IRA [15]. Valenti et al. confirmed the staged PCI of CTO in the non-IRA's role in improved cardiac survival at a 3-year follow-up in 1911 AMI patients [16]. Similarly, other 1-year follow-up studies or retrospective studies agreed to the benefits of successfully staged revascularization of CTO in the non-IRA for AMI patients [8, 17]. However, the multicentered EXPLORE study, the only randomized controlled trial that evaluated 302 patients with STEMI through a mean follow-up of 3.9 years, found no reduction of long-term MACCE [18]. Finally, Tong et al. carried out a meta-analysis of 1083 AMI patients, and concorded revascularization of CTO in the non-IRA was associated with a lower mortality rate at a mean follow-up of 36 months [19].

As for our findings, while most of our results conform with the previous studies, including the significantly lower LVEF, higher mortalities, and increased revascularization rates in the CTO group, some results differ. We discovered CTO in the non-IRA is an independent predictor of 11-year MACCE in male AMI individuals and male NSTEMI individuals, instead of the entire AMI population. We interpret three possible explanations: (1) we only enrolled AMI patients who had received successful emergent revascularization of IRA and excluded patients with cardiogenic shock art admission since we believe unsuccessful PCI and cardiogenic shock at presentation would contribute to the long-term MACCE, which could muddle the potential effects of CTO. (2) Our 11-year mortality rate is relatively lower than the previous studies, which corroborates with our enrollment process and indicates the relatively benign nature of our study population. A longer follow-up study of our population would witness increased mortalities and might generate different results. (3) Our study showed NSTEMI group had significantly decreased LVEF, increased episodes of previous MI, increased prevalence of the three-vessel disease, and predominantly type C lesions compared to STEMI patients. Although the prevalence of CTO in the non-IRA, in-hospital MACCE, and primary endpoints were similar between two groups, coronary atherosclerosis in NSTEMI patients was more severe. Furthermore, CTO in the non-IRA could exert more damage to the myocardium in this population, which could explain its independent predictive role of long-term MACCE in this subpopulation. (4) Only 60 patients received recanalization of CTO in the non-IRA. The long-term prognosis would be better if more patients had the CTO revascularized.

Moreover, our study revealed that AMI patients with CTO in the non-IRA had a significantly higher prevalence of type C lesions in the IRA, even though the risk factors were similar between the two groups. We think this unique finding indicates a more severe state of coronary atherosclerosis in patients with CTO in the non-IRA, which could explain the increased in-hospital and long-term mortalities. We also identified the subgroups with the three-vessel disease or left main plus three-vessel disease were at significantly higher risks of MACCE in the CTO group. Last but not least, due to the CTO's chronic effect of worsened myocardial ischemia, CTO's predictive role of long-term mortality in the NSTEMI patients could be explained by the more severe state of coronary atherosclerosis in this subpopulation, which was indicated by our study. Likewise, our results also showed that patients with MVD were at increased risk of mortalities in the CTO subgroup, which indicates that aggressive interventions should be employed for these high-risk subgroups.

Compared with the studies mentioned above, the present study merits the following aspects. (1) By far, our study is the most extensive follow-up study focusing on the association between CTO in the non-IRA and prognosis of AMI patients. The unprecedented 11-year follow-up data could provide valuable information for understanding the long-term prognostic role of CTO in the non-IRA. (2) Our sample size is the largest among the observational studies above, which makes our results more concrete. (3) Our dropout rate is 1.6% during the average follow-up length of 134.3 months. It is even lower than the relatively short follow-up studies above, which states our follow-up data's reliability. (4) The prevalence of CTO in the non-IRA and the baseline characteristics are similar to the previous studies, which could rule out the possibility of selection bias.

### 4.1. Limitations

Our study has several limitations. First, regardless of the most prolonged follow-up duration and large sample size, our study is single-center oriented. Second, as mentioned above, we excluded patients with cardiogenic shock or unsuccessful emergent revascularization. These two limits will decrease the external validity. However, our screening process aims to elucidate the effect of CTO. Third, nearly 84% of our study population were male. Although this might not reflect the real world, this phenomenon did prevail across other previous studies [2, 6, 7, 11, 12, 20]. And our result showed the predictive capability of CTO in the non-IRA only in the male subgroup. Fourth, our study could be more thorough if we recorded the syntax score for each patient. Last but not least, for the dual antiplatelet regimen, we only prescribe aspirin with clopidogrel or ticagrelor, without the choice of prasugrel, whereas other trials have suggested conflicting findings between prasugrel and ticagrelor for treating AMI patients [21, 22].

## 5. Conclusions

Our prospective observational study discovered that CTO in the non-IRA was associated with increased MACCE and lower survival rates in AMI patients. It was also an independent predictor of 11-year mortalities in male AMI patients. The subgroup of MVD in the CTO group was at pronounced risks of MACCE and mortality.

## Figures and Tables

**Table 1:** Baseline and angiographic characteristics of patients with STEMI or NSTEMI.

Variables		NSTEMI group *N* = 630	STEMI group *N* = 1652	*P* value	All *N* = 2282
Age		57.7 ± 10.02	55.0 ± 10.49	<0.001	
Male		504 (80.0%)	1420 (85.9%)	<0.001	1924 (84.3%)
Prior MI		303 (48.1%)	567 (34.3%)	<0.001	870 (38.1%)
Prior PCI		66 (10.5%)	111 (6.7%)	0.002703	177 (7.7%)
Prior stroke		28 (4.4%)	57 (3.5%)	0.2623	85 (3.7%)
Diabetes		140 (22.2%)	323 (19.5%)	0.1562	463 (20.3%)
Hypertension		335 (53.2%)	804 (48.7%)	0.05426	1139 (49.9%)
Hyperlipidemia		264 (41.9%)	711 (43.0%)	0.6245	975 (42.7%)

Smoking habits	No smoking history	336 (44.5%)	845 (51.2%)	<0.001	1211 (53.1%)
	Current smoker	229 (48.1%)	761 (64.1%)		990 (43.4%)
	Cessation of smoking	35 (7.4%)	46 (3.9%)		81 (3.5%)

LVEF estimated by echocardiography		59.4 % ± 8.48%	57.4% ± 8.52%	<0.001	57.9 (8.56%)

Number of vessels affected	Single-vessel disease	94 (15.7%)	439 (28.2%)	<0.001	533 (23.7%)
	Two-vessel disease	147 (24.6%)	479 (30.8%)		626 (27.9%)
	Three-vessel disease	348 (58.3%)	575 (37.0%)		923 (41.1%)
	Left main plus three-vessel disease	8 (1.3%)	61 (3.9%)		69 (3.1%)

Type of IRA's lesion	Type				
	A	21 (3.3%)	66 (4.0%)	0.2047	87 (3.8%)
	B1	63 (10.0%)	208 (12.6%)		271 (11.8%)
	B2	145 (23.0%)	355 (23.9%)		540 (23.6%)
	C	401 (63.7%)	982(59.4%)		1383 (60.7%)

CTO in the non-IRA		165 (26.2%)	462 (28.0%)	0.3697	627 (27.6%)
In-hospital MACCE		14 (2.2%)	41 (2.5%)	0.7177	55 (2.4%)
In-hospital recurrent AMI		6 (1.0%)	25 (1.5%)	0.3007	31 (1.4%)
In-hospital TLR		10 (1.6%)	27 (1.6%)	1	37 (1.6%)

In Table 1, we showed that patients with NSTEMI were younger, were predominantly male, were more active smokers, and had relatively lower left ventricular ejection fraction (LVEF) compared to patients with STEMI.

**Table 2:** Baseline and angiographic characteristics of patients with CTO or without CTO.

Variable		CTO group (*N* = 629)	No-CTO group (*N* = 1653)	*P* value
Age		55.6 ± 10.75	55.8 ± 10.66	0.6307
Male		523 (83.4%)	1397 (84.7%)	0.4832
Prior MI		263 (41.9%)	605 (36.7%)	0.02107
Prior PCI		38 (6.1%)	138 (8.4%)	0.06552
Stroke		18 (2.9%)	67 (4.1%)	0.1802
Diabetes		115 (18.3%)	347 (21.0%)	0.1522
Hypertension		323 (51.5%)	814 (49.4%)	0.359
Hyperlipidemia		254 (40.5%)	720 (43.7%)	0.1745

Smoking habit	No smoking history	335 (53.4%)	871 (52.8%)	0.2778
	Current smoker	276 (44.0%)	713 (43.2%)	
	Cessation of smoking	16 (2.6%)	65 (3.9%)	

LVEF estimated by echocardiography		56.4% ± 8.76%	58.5% ± 8.55%	<0.001

Number of vessels affected	Single-vessel disease	0 (0%)	401 (26.0%)	<.0001
	Two-vessel disease	194 (30.9%)	475 (30.8%)	
	Three-vessel disease	354 (56.3%)	612 (39.7%)	
	Left main plus three-vessel disease	58 (9.2%)	55 (3.6%)	

Type of IRA's lesion				
	A	1 (0.2%)	85 (5.2%)	<0.001
	B1	21 (3.3%)	250 (15.2%)	
	B2	94 (15.0%)	445 (27.0%)	
	C	510 (81.3%)	869 (52.7%)	

In-hospital MACCE		25(4.0%)	30 (1.8%)	0.002621
In-hospital recurrent AMI		8 (1.3%)	23 (1.4%)	0.827
In-hospital TLR		21 (3.3%)	16 (1.0%)	<0.001

In Table 2, we showed that patients with CTO in the non-IRA had significantly lower LVEF, increased previous MI episodes, increased prevalence of three-vessel disease, and predominantly type C lesions in the IRA compared to patients without CTO at baseline.

**Table 3:** Primary outcomes for CTO group and no-CTO group.

MACCE	CTO group (*N* = 629	No-CTO group (*N* = 1653)	Total (*N* = 2282)	*P* value
Death from cardiovascular causes	43 (6.8%)	82 (5.0%)	125 (5.5%)	0.0778
Recurrent AMI	45 (7.2%)	112 (6.8%)	157 (6.9%)	0.7461
Stroke	18 (2.9%)	30 (1.8%)	48 (2.1%)	0.1188
TLR	127 (20.2%)	270 (16.3%)	397 (17.4%)	0.0292
Total MACCE	191 (30.4%)	402 (24.3%)	593 (26.0%)	0.003468

In Table 3, we showed that CTO group had significantly increased primary endpoint rates compared to the group without CTO.

**Table 4:** Multivariable regression model of all-cause MACCE during the entire follow-up period in the whole study population (only showing CTO in the non-IRA and other variables with statistical significance).

Variable	Coefficient	Standard error	HR	95% CI lower limit	95% CI upper limit	*P* value
CTO in the non-IRA	−0.23471	0.137333	0.790799	0.604182	1.035057	0.087438
LVEF by echocardiography	−0.01565	0.005328	0.984467	0.974241	0.994801	0.0033
In-hospital MACCE	0.615082	0.23046	1.849808	1.177493	2.905996	0.007609
Two-vessel disease	0.300066	0.132752	1.349948	1.040683	1.75112	0.023799
Three-vessel disease	0.463517	0.123943	1.589655	1.246815	2.026767	0.000184

In Table 4, we discovered in-hospital MACCE, two-vessel disease, and three-vessel disease were independent predictors of 11-year MACCE in the entire study population. LVEF remained as a protective factor for survival.

**Table 5:** Multivariable regression model of MACCE during entire follow-up period in the male study population (only showing CTO in the non-IRA and other variables with statistical significance).

Variable	Coefficient	Standard error	HR	95% CI lower limit	95% CI upper limit	*P* value
CTO in the non-IRA	0.245562	0.094893	1.278339	1.061385	1.53964	0.010885
Age	0.009938	0.004323	1.009988	1.001465	1.018582	0.021591
LVEF by echocardiography	−0.01802	0.005437	0.98214	0.971731	0.992662	0.001025
In-hospital MACCE	0.577263	0.228156	1.781157	1.138926	2.785535	0.020204
In-hospital TLR	0.630757	0.262189	1.879032	1.12398	3.141301	0.028655
Three-vessel disease	0.269655	0.133949	1.309513	1.007145	1.702658	0.044102

In Table 5, in the male population, we identified that CTO, age, in-hospital MACCE, in-hospital TLR, and three-vessel disease were independent predictors of 11-year MACCE. LVEF remained as a protective factor.

## Data Availability

The data are securely available from the corresponding author upon request by email.

## References

[1] Claessen B. E., Dangas G. D., Weisz G. (2012). Prognostic impact of a chronic total occlusion in a non-infarct-related artery in patients with ST-segment elevation myocardial infarction: 3 year results from the HORIZONS-AMI trial. *European Heart Journal*.

[2] Ariza-Solé A., Teruel L., di Marco A. (2014). Prognostic impact of chronic total occlusion in a nonculprit artery in patients with acute myocardial infarction undergoing primary angioplasty. *Revista Española de Cardiología (English Edition)*.

[3] Råmunddal T., Hoebers L. P., Henriques J. P. S. (2016). Prognostic impact of chronic total occlusions: a report from scaar (Swedish coronary angiography and angioplasty registry). *Journal of American College of Cardiology: Cardiovascular Interventions*.

[4] Saad M., Stiermaier T., Fuernau G. (2018). Impact of chronic total occlusion in a non-infarct-related coronary artery on myocardial injury assessed by cardiac magnetic resonance imaging and prognosis in ST-elevation myocardial infarction. *International Journal of Cardiology*.

[5] Moreno R., Conde C., Perez-Vizcayno M. J. (2006 Jan). Prognostic impact of a chronic occlusion in a noninfarct vessel in patients with acute myocardial infarction and multivessel disease undergoing primary percutaneous coronary intervention. *Journal of Invasive Cardiology*.

[6] Lesiak M., Cugowska M., Araszkiewicz A. (2017). Impact of the presence of chronically occluded coronary artery on long-term prognosis of patients with acute ST-segment elevation myocardial infarction. *Cardiology Journal*.

[7] Tajstra M., Gasior M., Gierlotka M. (2012). Comparison of five-year outcomes of patients with and without chronic total occlusion of noninfarct coronary artery after primary coronary intervention for ST-segment elevation acute myocardial infarction. *The American Journal of Cardiology*.

[8] Deng J., Wang X., Shi Y., Zhao X., Han Y. (2018). Prognostic value of the age, creatinine, and ejection fraction score for non-infarct-related chronic total occlusion revascularization after primary percutaneous intervention in acute ST-elevation myocardial infarction patients: a retrospective study. *Journal of Interventional Cardiology*.

[9] Mozid A. M., Mohdnazri S., Mannakkara N. N. (2014). Impact of a chronic total occlusion in a non-infarct related artery on clinical outcomes following primary percutaneous intervention in acute ST-elevation myocardial infarction. *Journal of Invasive Cardiology*.

[10] Mizuguchi Y., Takahashi A., Hashimoto S. (2015). Impact of the presence of chronic total occlusion in a non-infarct-related coronary artery in acute myocardial infarction patients. *International Heart Journal*.

[11] Gierlotka M., Tajstra M., Gąsior M. (2013). Impact of chronic total occlusion artery on 12-month mortality in patients with non-ST-segment elevation myocardial infarction treated by percutaneous coronary intervention (from the PL-ACS Registry). *International Journal of Cardiology*.

[12] Lee J. H., Park H. S., Ryu H. M. (2012). Impact of multivessel coronary disease with chronic total occlusion on one-year mortality in patients with acute myocardial infarction. *Korean Circulation Journal*.

[13] O’Connor S. A., Garot P., Sanguineti F. (2015). Meta-analysis of the impact on mortality of noninfarct-related artery coronary chronic total occlusion in patients presenting with ST-segment elevation myocardial infarction. *The American Journal of Cardiology*.

[14] Yoshida R., Ishii H., Morishima I. (2019). Prognostic impact of recanalizing chronic total occlusion in non-infarct related arteries on long-term clinical outcomes in acute myocardial infarction patients undergoing primary percutaneous coronary intervention. *Cardiovascular Intervention and Therapeutics*.

[15] Choi I. J., Koh Y.-S., Lim S. (2016). Impact of percutaneous coronary intervention for chronic total occlusion in non-infarct-related arteries in patients with acute myocardial infarction (from the COREA-AMI registry). *The American Journal of Cardiology*.

[16] Valenti R., Marrani M., Cantini G. (2014). Impact of chronic total occlusion revascularization in patients with acute myocardial infarction treated by primary percutaneous coronary intervention. *The American Journal of Cardiology*.

[17] Park J. Y., Choi B. G., Rha S.-W. (2018). Chronic total occlusion intervention of the non-infarct-related artery in acute myocardial infarction patients. *Coronary Artery Disease*.

[18] Elias J., van Dongen I. M., Råmunddal T. (2018). Long-term impact of chronic total occlusion recanalisation in patients with ST-elevation myocardial infarction. *Heart*.

[19] Tong J., Yu Q., Li C., Shao X., Xia Y. (2018). Successful revascularization of noninfarct related artery with chronic total occlusion among acute myocardial infarction patients. *Medicine*.

[20] Zhang H.-P., Zhao Y., Li H. (2016). Impact of chronic total occlusion in a noninfarct-related artery on clinical outcomes in patients with acute ST-elevation myocardial infarction undergoing primary percutaneous coronary intervention. *Medicine*.

[21] Aytekin A., Ndrepepa G., Neumann F.-J. (2020). Ticagrelor or prasugrel in patients with ST-segment-elevation myocardial infarction undergoing primary percutaneous coronary intervention. *Circulation*.

[22] Krishnamurthy A., Keeble C., Anderson M. (2019). Real-world comparison of clopidogrel, prasugrel and ticagrelor in patients undergoing primary percutaneous coronary intervention. *Open Heart*.

